# Static and Dynamic Factors Promoting Resilience following Traumatic Brain Injury: A Brief Review

**DOI:** 10.1155/2015/902802

**Published:** 2015-08-04

**Authors:** Jessica N. Holland, Adam T. Schmidt

**Affiliations:** Department of Psychology and Philosophy, Sam Houston State University, Campus Box 2447, Huntsville, TX 77341, USA

## Abstract

Traumatic brain injury (TBI) is the greatest contributing cause of death and disability among children and young adults in the United States. The current paper briefly summarizes contemporary literature on factors that can improve outcomes (i.e., promote resilience) for children and adults following TBI. For the purpose of this paper, the authors divided these factors into static or unmodifiable factors (i.e., age, sex, intellectual abilities/education, and preinjury psychiatric history) and dynamic or modifiable factors (i.e., socioeconomic status, family functioning/social support, nutrition, and exercise). Drawing on human and animal studies, the research reviewed indicated that these various factors can improve outcomes in multiple domains of functioning (e.g., cognition, emotion regulation, health and wellness, behavior, etc.) following a TBI. However, many of these factors have not been studied across populations, have been limited to preclinical investigations, have been limited in their scope or follow-up, or have not involved a thorough evaluation of outcomes. Thus, although promising, continued research is vital in the area of factors promoting resilience following TBI in children and adults.

## 1. Introduction

Traumatic brain injury (TBI) is a significant contributor to mortality and morbidity in children and adults throughout the world [1, 2]. There are over one million new cases of TBI each year in the United States alone and together these account for over 7.6 billion dollars in medical costs and other expenditures [3]. However, the factors influencing positive outcomes and promoting resilience following these potentially devastating injuries are not completely understood. For example, a 2009 study by Fay and colleagues [4] found that four years after injury, well over half of the children sustaining a severe TBI had intact skills in at least three of four broad areas of psychosocial and cognitive functioning (e.g., neuropsychological functioning, behavioral functioning, adaptive skills, and academic skills). Similarly, another study by McCauley et al. [5] found that higher levels of resiliency and decreased depressed mood are correlated to decreased postinjury anxiety and postconcussive symptoms in adults.

In psychology, the process by which individuals exhibit positive adaptation following exposure to a hardship is known as resilience [6]. The concept of resilience has also been applied, although somewhat sparingly, to the process of improved recovery trajectories following a TBI [5, 7, 8]. Researchers are now focusing on identifying what factors allow some people to “beat the odds” by overcoming the impairments caused by their injury (i.e., exhibit resilient functioning) [9]. In the current paper, we briefly review the factors that have been related to positive outcomes/resilience following a TBI in children and adults. The focus on protective verses risk factors is intentional as we wish to highlight variables that researchers and clinicians working with individuals following a TBI may consider when evaluating and designing rehabilitation programs. Further, by couching our discussion in terms of factors promoting recovery we intend to emphasize the potential for positive outcomes following TBI, spur future research into these and other factors not yet identified, and facilitate an understanding of the mechanisms that promote resilience and neural plasticity following a TBI.

For the purpose of this paper, we divided the factors that influence outcomes into two groups: static factors (i.e., nonmodifiable factors that are a consequence of the individual's traits or demographics and cannot be changed through interventions) and dynamic factors (i.e., modifiable factors that could be addressed through interventions). Although the static factors cannot be changed through intervention, they are important contributors for clinicians to keep in mind when treating patients after an injury. The most thoroughly researched of these include age, biological sex, intellectual ability/intelligence, and preinjury psychiatric history [2, 4, 9]. Conversely, the dynamic factors that may be amenable to intervention and could potentially be incorporated into rehabilitation programs and treatment approaches include access to rehabilitation services, family functioning/social support, nutrition, and exercise [2, 4, 5, 7–9]. Importantly, each of these factors could be the subject for an entire integrated review. However, the purpose of the current paper is to provide a succinct overview and synopsis of the recent research on factors promoting recovery following TBI. As such, whenever possible, we have concentrated our discussion on emerging trends and articles published in the past decade.

## 2. Static Protective Factors

### 2.1. Age

Age is a significant factor when evaluating the recovery trajectory of a patient who has sustained a TBI [10–12]. Due to the increased plasticity of the brain in younger people, it was once believed that the younger the person at the time of injury, the better the postinjury outcome [10, 11]. This was referred to as the “Kennard” principle [12]. Subsequent investigations revealed an opposite pattern [13, 14]. That is, younger children show attenuated recovery patterns compared to older children and younger adult populations due to their incomplete neurological development [15]. In particular, brain regions such as the prefrontal cortex (and frontal lobes more generally) undergo a very protracted period of development making them exquisitely vulnerable to the effects of early brain insults [16].

Anderson et al. [17] analyzed how the developing brain is affected by a TBI through analyzing children's cognitive outcomes directly after and 12 months and 30 months after injury. The children were divided into two groups based on age, younger children (3–7 years) and older children (8–12 years); the children were also grouped by their injury type (i.e., mild, moderate, and severe). Results indicated that younger children who sustained a more severe insult displayed attenuated cognitive recovery patterns compared to older participants who endured the same injury. Karver et al. [18] examined the effects of age at injury on the prevalence of behavioral deficits in children who sustained a TBI compared to children who sustained an orthopedic injury (OI). They found that children who sustained a TBI at an earlier age had higher parent-reported symptoms than children who sustained their injury at an older age. Younger children also have more social deficits following a TBI. Ryan et al. [19] found that individuals sustaining a TBI during childhood had poorer emotional perception than healthy controls, suggesting that deficits in emotional perception experienced during childhood may continue into adulthood for TBI survivors. These studies and similar investigations illustrate early neurological damage results in more deficits and a decreased likelihood of full recovery across domains of functioning. Conversely, they suggest that individuals older at the time of their initial injury may fair better and exhibit a more positive outcome [17, 18].

Although not as many studies exist with adults, the picture appears more complex. There is some evidence that a similar effect may be observed with regard to development of depression following a closed head injury in adults. Specifically, studies involving adult participants suggest that older adults who sustained a TBI were less likely to develop major depression when compared to their younger adult peers [8]. They compared older patients who endured a mild TBI (i.e., over 60 years old) to younger patients who also suffered from a mild TBI (i.e., under 60 years old) and found that the older patients seem to be relatively resilient to major depression after their injury. The authors hypothesized that depression may be less prevalent in general within the population of older adults included in the study. They argue that more research is needed in the future to understand the direct correlation between these findings. Conversely, a recent study by Schmidt et al. [20] suggested that relatively older adults who sustained a mild TBI exhibited a greater number of sleep difficulties over the first three months after injury compared to adolescent and younger adults. Taken together, findings in children clearly suggest that older age at injury is associated with better outcomes following a TBI [8, 18, 19]. Although not as robust in adults, there is some evidence, at least with regard to depression, that a similar pattern may emerge following mild injuries [8]. Nonetheless, there have been few studies explicitly examining the relationship of age to specific outcomes in adult populations (e.g., studies comparing younger to middle-aged to older adults) [8].

### 2.2. Sex

Multiple studies suggest that biological sex is another static factor that affects the recovery pattern following a TBI [21–23]. According to the Centers for Disease Control, men are three times more likely to die from a TBI than women [1], and much research has focused on how sex may influence recovery following these injuries [21, 23]. Many studies have indicated that women have a better prognosis following closed head injuries [24], and gaining a thorough understanding of the determinants of this effect may lead to improvements in existing treatments or the development of entirely new therapies [24].

Most of the studies investigating the mechanisms behind this effect have focused on the role of progesterone as a protective factor [25]. The precise mechanism that progesterone plays in the brain following a TBI remains unknown [26]; however, it has been found to decrease edema [27], guard the blood brain barrier [28], decrease inflammatory responses [29], increase progenitor cell levels [30], and regulate calcium signaling [31] following the injury. Interest in progesterone as a possible protective factor following TBI began after clinicians noticed that females appeared to have relatively better outcomes compared to males with injuries of similar severity [24, 25]. Progesterone is hypothesized to improve outcomes in females by helping to reduce inflammation and apoptosis within the hippocampus in the acute and postacute period following an injury [32].

Although numerous studies suggested that women have an improved trajectory of recovery compared to men, following TBI [23–25], Renner et al. [33] argued for an alternate explanation of these findings. Specifically, the authors suggested that males and females differed on the severity of the injuries they sustained and that this difference accounted for generally better outcomes of females after a TBI. Moreover, a few studies have indicated that males recover better from concussions compared to females [34]. Regardless of the mechanism, most of the available evidence suggested a difference in outcomes for males and females following a TBI with females generally showing a more accelerated trajectory of recovery [21–24].

### 2.3. Intellectual Abilities/Education

Although level of education may be somewhat of a dynamic factor, the decision was made by the authors to categorize it as static because few studies have examined the specific role of postinjury education in promoting recovery after a TBI. The neurological damage that occurs during and after a TBI, specifically damage to the temporal and frontal lobes, frequently disrupts intellectual and cognitive abilities [2]. Complex reasoning and problem solving, executive functions, memory, attention, and psychosocial functioning are cognitive domains often compromised following a TBI and are significantly involved in intellectual abilities and educational attainment [2, 35]. Research indicates that individuals with higher preinjury intellectual and cognitive abilities exhibit a positive recovery trajectory (i.e., return to baseline or near baseline functioning) when compared to their lower preinjury functioning peers [36, 37]. Further, data suggest that individuals with higher preinjury intellectual abilities demonstrate fewer long-term cognitive deficits during the postinjury recovery period [36, 37], a finding indicative of greater cognitive reserve in these individuals.

Most research in this area has focused on children because of the impact of early injuries on intellectual and academic development [35]. McNally et al. [37] examined how injury characteristics (i.e., type of injury) versus noninjury characteristics (i.e., preinjury symptoms and intelligence) predicted postconcussive symptoms in children who had a mild TBI compared to children sustaining an OI. They found that children with higher preinjury intellectual abilities demonstrated fewer physical postconcussive symptoms, whereas children with lower preinjury intellectual abilities displayed more postconcussive symptoms across domains. The authors maintained that their results demonstrated that children with lower preinjury cognitive abilities may not have the resources to adequately cope with neurological traumas especially when compared to their more intellectually precocious peers [37].

Although many children sustaining a TBI require special education following their injury [38], some studies suggested that preinjury academic skills are related to postinjury functioning. For example, Catroppa and Anderson [36] analyzed preinjury intellectual functioning on academic success following pediatric TBI 6, 12, and 24 months after injury. Results indicated that reading accuracy, spelling, arithmetic, and listening comprehension at 24 months after injury were best predicted by the child's preinjury ability in these specific domains. The authors suggested that these cognitive abilities are established early on in the child's life, prior to the injury, and may serve as a significant protective factor, buffering higher-performing children from the negative consequences following a TBI [36]. These studies indicate that higher preinjury cognitive functioning facilitates recovery of skills after injury and leads to a better long-term outcome. Importantly, there is a significant gap in our understanding of how post-injury educational attainment and programming influences long-term cognitive recovery and academic progress following a TBI.

### 2.4. Preinjury Psychiatric History

Psychiatric sequelae are some of the most pervasive problems for individuals who have sustained a TBI [2]. Often, the psychiatric sequelae can be more devastating than the neurological and physical trauma the person initially endures [39]. Many people suffer from posttraumatic stress disorder (PTSD), depression, anxiety disorders, substance disorders, and various personality disorders after sustaining a TBI [40]. Although the precise causative factors for each specific psychiatric disorder following a TBI may vary, there is evidence that preinjury psychiatric history is an important variable in a person's postinjury emotional adjustment and recovery of function following their trauma [5, 41].

A 2010 study examined the role of preinjury psychiatric status by using a multivariate analysis to study 100 community-based participants, from 19–74 years of age, who sustained a TBI [41]. The authors found that the preinjury psychiatric history of the participants was a substantial risk factor for developing a psychiatric disorder following their injury. Specifically, 65% of participants were diagnosed with at least one psychiatric disorder after injury, and a significant number of these cases were novel psychiatric disorders. Consequently, 46% of participants were diagnosed with postinjury depression, and of those diagnoses 72% (33 participants) were new-onset cases with depression arising after injury. The study also found that 38% of participants were diagnosed with postinjury anxiety, and of those diagnoses 74% (28 participants) were novel disorders. This research indicated that not only there was a correlation between existing preinjury and postinjury psychiatric disorders but also there was a significant correlation between preinjury psychiatric disorders and novel psychiatric disorders following a TBI [41].

Researchers have explored how preinjury mood influences the recovery trajectory of participants following a mild TBI [5]. In this study, researchers examined how depressed mood influenced anxiety and postconcussive symptom severity the day of injury and one week and one month after injury. Their results indicated that preinjury mood was significantly and positively correlated with anxiety and postconcussive symptom severity, suggesting the more depressive symptoms an individual exhibits prior to their injury, the worse the trajectory of their recovery in terms of postconcussive symptoms [5]. Taken together, various studies suggest that preinjury psychiatric functioning can influence the development of novel psychiatric functioning after injury and may thus play a pivotal role in promoting resilience following TBI [5, 41].

The above discussion has focused on static factors that are generally not modifiable (although as noted above, education does not fit neatly into either category). However, although it is important to understand the factors that may be stable/fixed and thus need to be accounted for by healthcare providers and researchers working with individuals sustaining a TBI, there are also a variety of dynamic factors that have been shown to positively influence recovery and can be potentially modified through intervention. The following discussion focuses on those variables that are considered dynamic and thus may be amenable to treatment.

## 3. Dynamic Protective Factors

### 3.1. Socioeconomic Status

Socioeconomic status (SES) is one of the most significant predictors of recovery following TBI [42]. Somewhat like education, SES could be considered to be both a static and a dynamic factor. However, the decision was made to place it in the dynamic category because much of the research in this area suggests that SES exerts its effect on recovery via indirect pathways such as access to appropriate follow-up medical care, adequate rehabilitation services, and educational training programs [2, 15, 19, 42, 43].

SES can influence the trajectory of recovery in a variety of domains including social cognition, emotion perception, behavior, adaptive abilities, and intellectual abilities [7, 19, 43]. Catroppa and Anderson [7] analyzed the intellectual capabilities of 70 children who sustained mild, moderate, and severe TBIs. They found that the best predictor of intellectual outcome following a TBI was socioeconomic status. Similar results were obtained using a much more basic cognitive paradigm (i.e., emotion recognition) [43]. These researchers found that family resources of the children in the TBI group were significantly related to an increased recovery of emotion processing skills (specifically, perception of emotional prosody). These results appeared unique to the TBI group; a similar pattern was not observed among the OI participants [43]. The authors speculated that this finding may represent increased access to rehabilitation services and/or reduced stress levels in those families with relatively more economic resources [43].

Similar results were found in a study conducted by Ryan et al. [19]. These researchers examined the relationship between SES and social cognitive skills in long-term, young-adult survivors of head injury. They compared young adult survivors with TBI to noninjured participants matched on age, sex, and SES. Results showed that young adults in the high-SES TBI group exhibited similar social cognition skills to noninjured high-SES participants and significantly better performance when compared to the low-SES TBI group [19]. Other studies have also indicated that SES is a significant contributor to other outcomes (i.e., memory, behavior, adaptive ability, reading, and spelling) following a severe TBI (see Anderson et al. [44] and Lajiness-O'Neill et al. [45]). It should be noted that some investigators have speculated that lower SES may increase the risk of TBI [46] potentially increasing the complications and economic burden of TBI for this portion of the population. Taken together, evidence indicates that SES is a major contributor to resilient function and long-term recovery following TBI across a number of cognitive and psychosocial domains. Although the mechanisms of this relationship have not been well-elucidated, it is plausible that access to intervention and rehabilitation services likely play a significant role in promoting more positive outcomes for individuals with higher SES backgrounds [19, 42–44].

### 3.2. Family Functioning/Social Support

The social support a person receives following TBI significantly influences their recovery trajectory [15, 47]. Family stress in particular has been linked to a number of cognitive and social outcomes, especially in children following an injury [44]. In one study, researchers examined the association of family functioning and academic performance in a group of children sustaining moderate TBI, severe TBI, or an OI [48]. Their results indicated that children sustaining a severe TBI who lived in a low-stress family environment exhibited an accelerated rate of academic recovery compared to children living in a high-stress family environment. Wade et al. [49] argued that families of children sustaining a severe TBI were more likely to experience greater stress when compared to families sustaining milder injuries. However, another research indicates that parental warmth serves as a protective factor following TBI, even in more severe injuries [50]. Wade and colleagues [50] examined the association between parental warmth and behavioral problems in children following a TBI. Results indicated children who experienced a severe TBI but were exposed to warm and responsive parenting demonstrated fewer behavioral problems, including internalizing difficulties, and ADHD symptoms compared to children who were exposed to less parental warmth following their injury. Further, this study determined that parental negativity exacerbated externalized behavior problems following a severe head injury.

In adult populations, social support and family functioning are less researched although a number of studies have addressed this issue [51, 52]. One study analyzed adults who suffered from a severe TBI and found that stronger social support was related to both decreased depression and increased well-being in adults who had sustained a TBI [53]. In addition, Groom et al. [54] examined the family members of individuals who sustained a TBI and discovered that increased neurobehavioral impairments (i.e., depression and deficits in pragmatics) were closely related to family dysfunction in adults following a TBI. In summary, research in both child and adult populations suggests that improved family functioning and increased social support facilitate protective factors following a TBI [47, 49–52].

### 3.3. Nutrition

Under normal circumstances, the brain is a highly metabolic, active organ [2, 11, 55]. This metabolic activity is further enhanced when the brain is in a state of repair following an acute injury [11, 55]. Consequently, the brain is vulnerable to inadequate energy input and deficits in specific micronutrients. This can result in a “brain energy crisis,” a state that hinders cell survival with implications for long-term cognitive function [55]. Given its high metabolic demands, adequate nutrition is an important protective factor that may help facilitate positive recovery following a TBI [55].

Recent studies suggest that nutrition during early life influences recovery from an acute brain injury [56]. Agrawal et al. [56] investigated the impact of pre- and perinatal nutrition on recovery following a subsequent brain injury. These investigators provided animals a pre- and perinatal diet that was either adequate or deficient in omega-3 fatty acids and then exposed the groups to a concussive injury once they reached maturity (about postnatal week 17). Animals in the deficient group who sustained a concussive injury exhibited more anxiety-like behaviors when compared to the group that had adequate pre- and perinatal nutrition. Within the concussed group exposed to the deficient diet, findings indicated decreases in adenosine monophosphate-activated protein kinase (AMPK) and peroxisome proliferator-activated receptor gamma coactivator 1-alpha (PGC-1*α*), which are both important in the formation of adenosine triphosphate (ATP) [56].

Other investigations have focused on the role of micronutrients following brain injury. In one study, Cope and colleagues [57] provided animals either a zinc deficient (5 ppm), zinc adequate (30 ppm), or zinc supplemented (180 ppm) diet for 4 weeks and then each animal underwent a concussive injury. Results revealed that zinc adequate and zinc supplemented animals demonstrated significantly less anxiety-like behaviors, fewer behaviors indicative of depression, and better spatial learning and memory skills when compared to the zinc-deficient group. In addition to micronutrients, TBIs require an increased demand for energy in the brain, creating an increased need for glucose [11, 55]. One study analyzed the effects of glucose following a controlled cortical impact (CCI) using a rodent model [58]. Results indicated that administration of a dose of glucose directly following the CCI significantly improved survival of cortical neurons. Taken together, these results illustrated the importance of adequate nutrition early in life in promoting positive outcomes following a subsequent brain injury [55–58].

Despite the promising findings using animal models, very few empirical studies have examined the role of nutrition in promoting recovery after TBI in human participants. A systematic review of the influence of metabolism and nutrition following TBI suggested a relationship between mortality and morbidity when patients were introduced to solid food following their injury [59, 60]. Although this may reflect the influence of injury severity, the authors argued that their finding was influenced by patients who were reintroduced to solid food earlier being better able to accommodate the increased metabolic rate and rapid protein breakdown that occur after a moderate or severe TBI. Another more recent systematic review concluded that, despite some encouraging preclinical animal studies, the numerous inconsistencies within human studies preclude definitive conclusions regarding the role of nutrition within TBI recovery [61]. These authors argued it is imperative that further research should be completed on human patients to determine if nutrient supplementation can provide the same types of neuroprotective effects for persons sustaining a closed head injury as suggested by animal models.

### 3.4. Exercise

In addition to its various long-term health benefits, exercise is another important dynamic factor that may influence recovery following a TBI [62–65]. In healthy animals and humans, exercise increases neurogenesis in the hippocampus and is associated with improvements in learning and memory [62]. Exercise is related to decreases in neuronal apoptosis following a TBI [63]. Using an animal model of TBI, Itoh and colleagues [63] divided animals into an exercise and nonexercise group. Results showed that the animals in the exercise group experienced less neuronal degeneration and apoptotic cell death in the brain region surrounding the injury location. Animals in the exercise group also showed improved outcomes in learning and memory as indicated by decreased swim times in the Morris Water Maze [63].

Although many of the studies examining the benefits of exercise following TBI have been conducted using animal models, a few investigations with human participants have been undertaken (see Schneider et al. [64] and Fogelman and Zafonte [65] for a review). Briefly, although these studies range in sophistication, scientific rigger, and generalizability [66], most data with human participants suggests that exercise may be a reasonable adjunct to treatment following a TBI and may facilitate positive outcomes. Specifically, low-intensity aerobic exercise has been demonstrated to have a positive impact following mild TBI [64]. Similarly, contrary to the traditional wisdom of rest for an extended period of time following mild TBI, Silverberg and Iverson [67] argue that more contemporary studies suggest that a brief period of rest followed by a relatively rapid return to noncontact activities including exercise is associated with better recovery across a wide range of populations. Nonetheless, despite these promising findings and encouraging evidence from animal studies, a major gap in our understanding of the protective role of exercise is a lack of well-controlled, rigorous investigations that examine the effect of exercise across TBI severity and across child and adult populations [66].

## 4. Summary and Future Directions

The interest on factors that influence recovery grew out of a desire to understand how and why certain people are able to rise above their experiences [4, 5, 68]. This brief review endeavored to explore the burgeoning literature that explores the various factors associated with positive recovery and resilience following a substantial closed head injury [5, 17, 26, 43, 56]. For the purpose of this review, these resilience promoting factors were defined as static (i.e., unmodifiable factors that cannot be altered by intervention such as age, sex, intellectual abilities/education, preinjury psychiatric history) and dynamic factors (i.e., modifiable factors that may be amenable to treatment socioeconomic status, family functioning/social support, nutrition, exercise). As stated in the text, the decision to place these factors into these categories was arbitrary but was intended to reflect the current stage of knowledge. For example, education was placed in the static category because of the lack of research on the influence of postinjury education on recovery [2]. Likewise, SES was placed in the dynamic category because of the potential to address the hypothesized mechanism of this variable (i.e., restricted access to rehabilitation and other support services) [2, 43]. Additionally, other variables such as the potential influence of genetic factors on TBI recovery were not included in this review because of the relative paucity of information regarding these factors [2]. Nonetheless, the influence of specific genes and gene-environment interactions on resilience following TBI appears to be a fruitful avenue for future investigations (see Graham et al. [69] for a brief discussion of this research).

Although there has been significant advancement in our understanding of factors that promote positive functioning and resilience following TBI, this brief review reveals substantial areas for future investigations. For example, the preponderance of research regarding the protective effects of age, intellectual abilities, and socioeconomic status following a TBI has been conducted with child participants. Therefore, more systematic investigations are needed to determine how these variables impact the recovery of adults across the age range and how these factors influence outcomes, such as return to work, parenting, and family functioning [2, 7–9, 17, 19]. Similarly, most of the research to date on factors such as nutrition and exercise has been conducted using animal models. Although these studies and the small number of investigations with human participants yield valuable insights, clinical studies (i.e., randomized, placebo-controlled, double-blind investigations) using larger samples of human participants are necessary to determine if nutrition and exercise are useful adjuncts to treatment and enhance positive outcomes following a TBI [59–61, 63–65].

This brief review was intended to highlight factors that may influence recovery and promote resilience after a TBI in order to provide both practitioners and researchers insights into those areas that may need to be considered both in future studies and when designing rehabilitation programs for TBI survivors and their families. By focusing on factors that promote positive as opposed to negative outcomes, we wished to illustrate that a substantial degree of long-term recovery is possible and may be accentuated by additional studies that take a positive adaptation approach to examining postinjury functioning. Further research into factors promoting recovery and resilience following TBI holds promise for improving interventions for a variety of neurologic populations. Additionally, future research can shed light on the neurobiological, behavioral, and psychosocial underpinnings of positive adaptation and the resilience process more generally. These insights are necessary to design treatments that address the needs of patients across multiple levels and build on the innate strengths of individuals and their families who directly experience the impact of these devastating injuries.
